# Supplementary material for: A Stretchable Expanded Polytetrafluorethylene-Silicone Elastomer Composite Electret for Wearable Sensor
Source: Nanomaterials (Basel). 2022 Dec 29;13(1):158. doi: 10.3390/nano13010158 (PMC9823660; doi:10.3390/nano13010158)
Supplement: Supplementary file 1 [file nanomaterials-13-00158-s001.zip › nanomaterials-2082576-supplementary.pdf]

Supporting Information

A Stretchable Expanded Polytetrafluorethylene-Silicone Elastomer Composite Electret for Wearable Sensor

Jianbo Tan [1], Kaikai Chen [1], Jinzhan Cheng [1], Zhaoqin Song [1], Jiahui Zhang [1], Shaodi Zheng [1,2,*], Zisheng Xu [1,2,*] and Shiju E [1,2]

[1] Key Laboratory of Urban Rail Transit Intelligent Operation and Maintenance Technology & Equipment of Zhejiang Province, College of Engineering, Zhejiang Normal University, Jinhua 321004, China; 1974179451@zjnu.edu.cn (J.T.); kirkchen@zjnu.edu.cn (K.C.); chengjinzhan@zjnu.edu.cn (J.C.); song021128@zjnu.edu.cn (Z.S.); zjh15327911239@zjnu.edu.cn (J.Z.); shiju.e@zjnu.edu.cn (S.E.)

[2] Jinhua Intelligent Manufacturing Research Institute, Jinhua 321004, China

* Correspondence: zhengs7118@zjnu.edu.cn (S.Z.); zishengxu@zjnu.edu.cn (Z.X.)

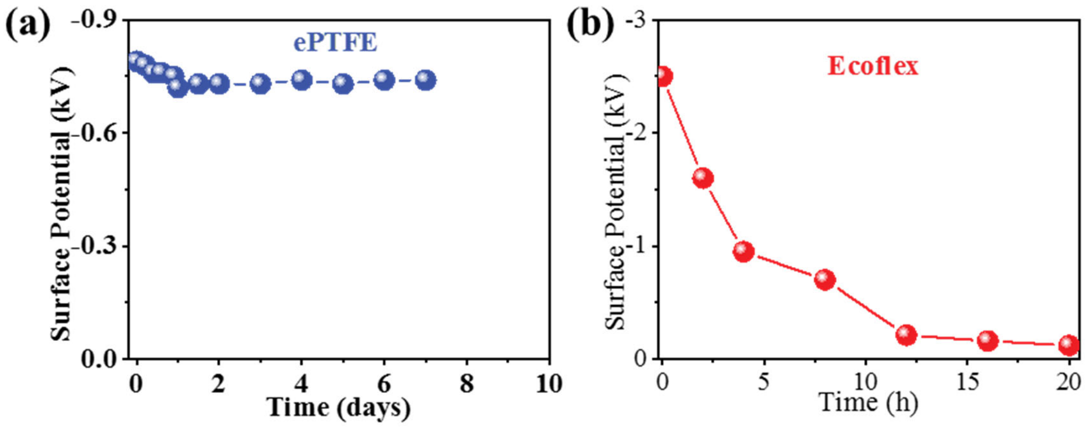

Fig.S1. Surface potential decay of (a) the ePTFE membrane and (b) Ecoflex film.

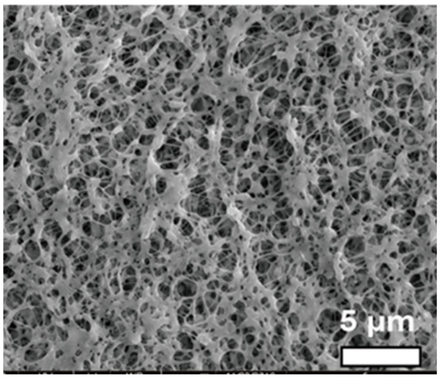

Fig.S2. SEM image of the pristine ePTFE membrane

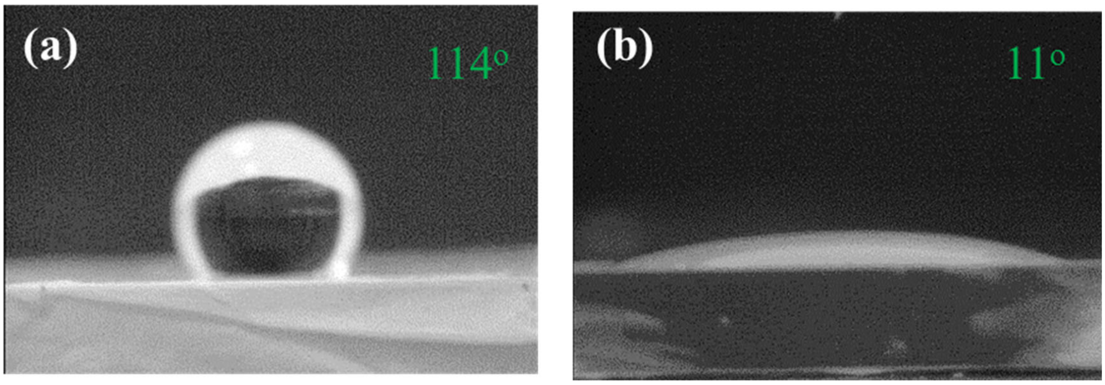

Fig.S3. Contact angles of water (a) and Ecoflex oilgomer (b) on the surface of ePTFE membrane.

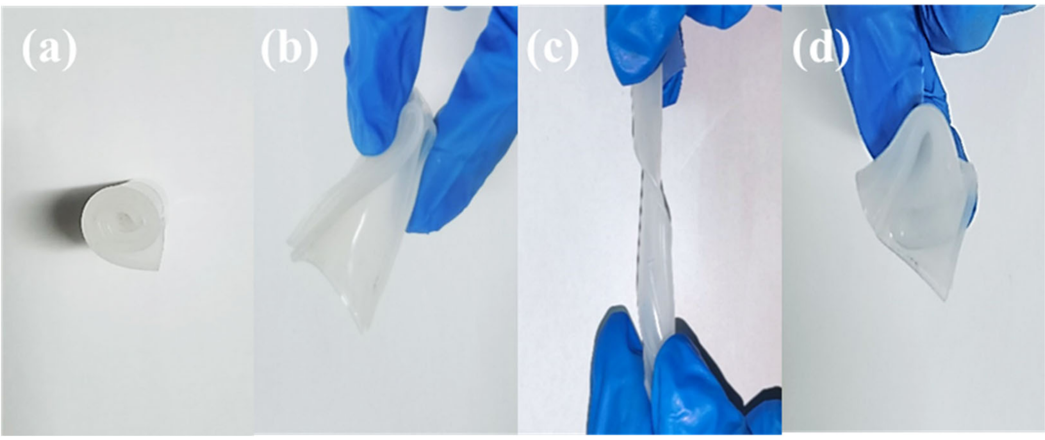

Fig.S4. Photos displaying superior flexibility of the electret composite under different deformation (a) rolling up, (b) folding, (c) twisting and (d) crumpling

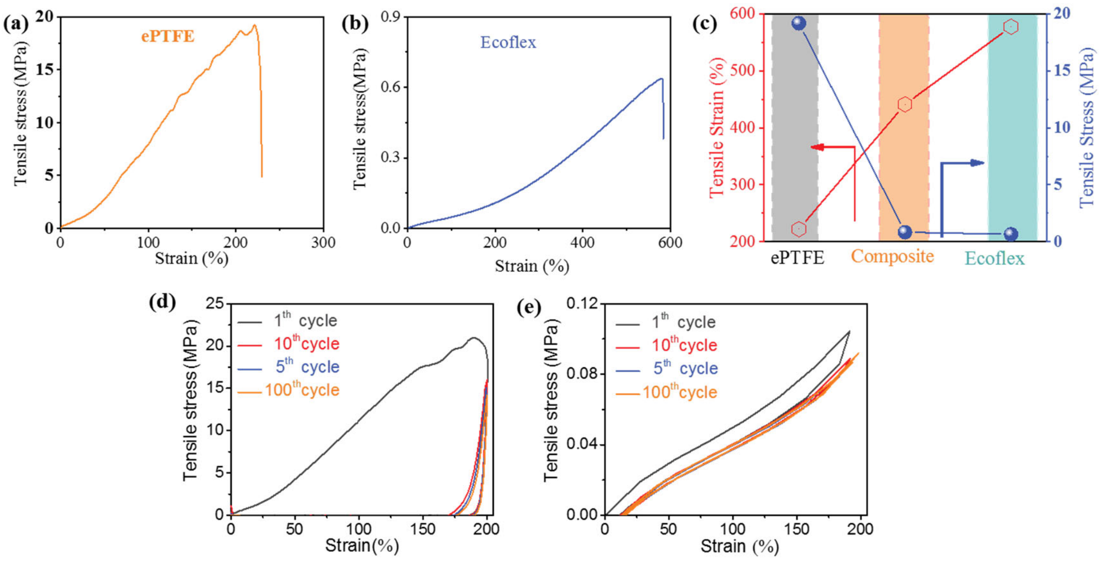

Fig.S5. Stress-strain curves of the ePTFE film (a) and Ecoflex (b) under uniaxial tensile test. (c)

Comparison of the tensile strain and stress of the ePTFE, Ecoflex and electret composite. Stress-strain curves of the ePTFE film (d) and Ecoflex (e) under cyclic tensile test (200% strain).

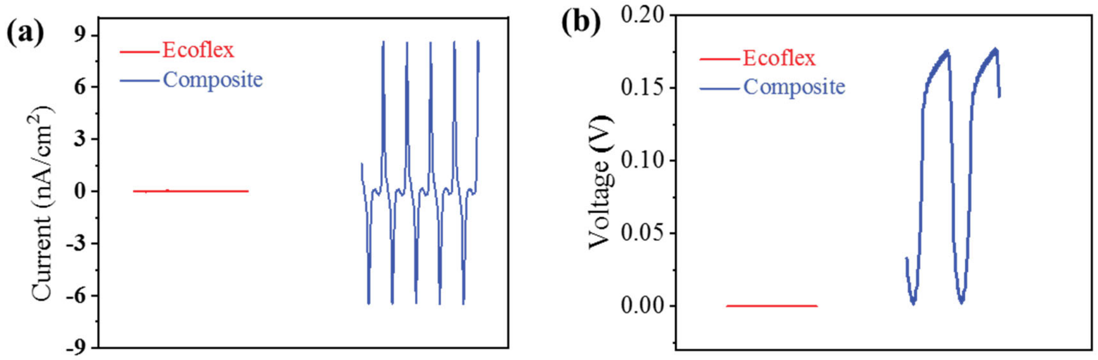

Fig.S6. The variation of output current (a) and voltage (b) of Ecoflex and electret composite under periodic tensile test.

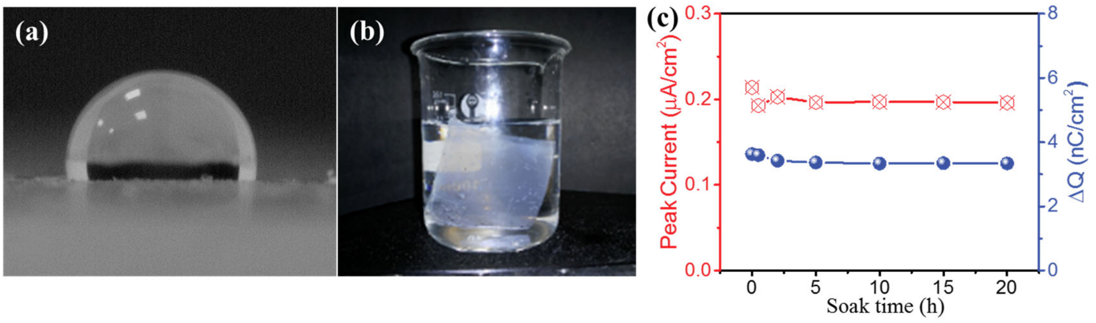

Fig.S7. (a)The water contact angle of the as-fabricated sensor. (b) The photo of the sensor soaked in the water. (c) The peak current and ΔQ of the sensor as a function of soak time.

Supplementary Note 1:

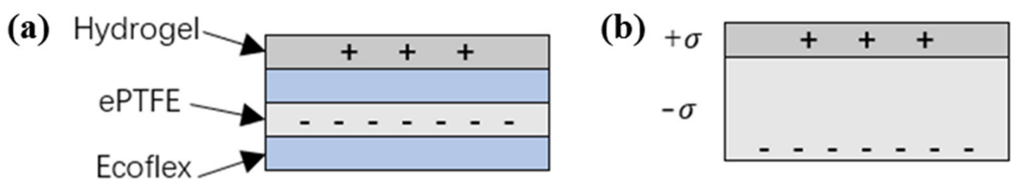

Fig.S8. (a) Basic structure diagram of stretchable electret. (b) Basic physical model of stretchable electret

The basic structure of the stretchable electret sensor is shown in Fig. S8a. The sensor is assembled by hydrogel electrode and electret composite with negative charges. According to

electrostatic induction and Gauss theorem, the electrode will induce opposite charges, namely positive charges. Thus, a physical model of the stretchable electret sensor can be established, as shown in Fig. S8b.

$$E = \frac{-\sigma}{\varepsilon_0 \varepsilon_r} \tag{1}$$

Where ε_0 and ε_r are the relative permittivity of the air and dielectric. In this article, the dielectric of a stretchable electret sensor is Ecoflex, and its relative dielectric constant is about 2.1. According to Kirchhoff's law, the output voltage of the stretchable electret sensor can be given by:

$$V = E \times d = \frac{-\sigma}{\varepsilon_0 \varepsilon_r} \times d \tag{2}$$

Where the d is the distance between the Ecoflex and the ePTFE. When the stretchable electret sensor is compressed by an external force, the distance between the ePTFE and the Ecoflex becomes smaller, resulting in a change in the output voltage of the stretchable electret sensor

$$V = \frac{-\sigma}{\varepsilon_0 \varepsilon_r} \times (d - \Delta d) \tag{3}$$

Where Δd is the variation in the thickness of Ecoflex film with an applied pressure. Based on the stress and strain formulas,

$$\Delta d = \frac{F}{sk} \times d \tag{4}$$

where F is the pressure applied externally to the stretchable electret sensor, s is the area of the stretchable electret sensor, k is the Young's modulus of Ecoflex, and the variation in the thickness of the Ecoflex film can be obtained with the known magnitude and area of the external force and Young's modulus. Thus, substitution of Eq. (4) into Eq. (3) yields:

$$V = \frac{-\sigma}{\varepsilon_0 \varepsilon_r} (d\text{-}\frac{F}{sk} \times d) \tag{5}$$

The Equation 5 is the relationship between the external force and the output voltage of the stretchable electret sensor under compression. Since the external force F is a time-varying quantity, the sensor output voltage V is also a time-varying quantity. Substituting the time variable into 5 yields:

$$V(t) = \frac{-\sigma}{\varepsilon_0 \varepsilon_r} (d\text{-}\frac{F(t)}{sk} \times d) \tag{6}$$

It can be seen from Equation 6 that the output voltage of the stretchable electret sensor under compression is related to the surface charge density of the ePTFE electret, the relative

dielectric constant and Young's modulus of Ecoflex, the actual area of the stretchable electret sensor and the external force changing with time.

When the stretchable electret is stretched by an external force, the distance between the ePTFE and the Ecoflex becomes smaller, and the surface charge density of the ePTFE electret decreases because the area becomes larger during stretching. In the case that the volume of the stretchable electret is constant, the multiple of the area increase is equal to the multiple of the thickness reduction. Therefore, it is assumed that the multiple of the area increase is m, and the multiple of the thickness reduction is also m. Substituting the m value into Equation 2, yields:

$$V = E_s \times d_s = \frac{-\sigma/m}{\varepsilon_0 \varepsilon_r} \times \frac{d}{m} = \frac{-\sigma}{\varepsilon_0 \varepsilon_r} \times \frac{d}{m^2} \quad (7)$$

$$m = \frac{F}{sk} \quad (8)$$

Combining Equations 7 and 8,

$$V = \frac{-\sigma}{\varepsilon_0 \varepsilon_r} \times \frac{d \times s^2 k^2}{F^2} \quad (9)$$

Therefore, the decrease of the surface charge density when the electret is stretched leads to a decrease in the output voltage.
